# Our Experience with Left-Sided Abomasal Displacement Correction via the Roll-and-Toggle-Pin Suture Procedure according to Grymer/Sterner Model

**DOI:** 10.5402/2011/572842

**Published:** 2011-12-27

**Authors:** T. Zadnik, R. Lombar

**Affiliations:** ^1^Clinic for Ruminants, Veterinary Faculty, University of Ljubljana, Cesta v Mestni log 47, 1000 Ljubljana, Slovenia; ^2^Veterinarska praksa Tenetiše, d.o.o., Tenetiše 80, 4204 Golnik, Slovenia

## Abstract

All over the world, and also in Slovenia, left-sided displacement of the abomasum (LDA) occurs most commonly in large-sized, high-producing adult dairy cows immediately after parturition. Yearly retrospective analyses of our ambulatory records showed significantly increased prevalence of LDA (2000 = 0.9%, 2010 = 3.7%), especially in cows after first parturition. Surgical replacement is now commonly practiced, and many techniques have been devised with emphasis on avoidance of recurrence of the displacement. Because of good results as recorded in the literature and encouragement of Keith E. Sterner, the author of this method, we want to try the right paramedian abomasopexy—Grymer/Sterner model. Since May 2009 till October 2011 109 cows from 46 farms were operated on because of LDA. As many as 44 (40.3%) were affected with LDA after first parturition. The analysis of successful procedure that was carried out 2 months after suture showed that 104 (95.4%) cows were cured. Only 5 (4.5%) cows died within 24 hours after surgery (4 cases of severe toxemia with hypokalemia and one case of acute abomasal hemorrhage were established). Our experience with Grymer/Sterner LDA transfixation sutures proved favorable. Because roll-and-toggle-pin suture technique is rapid and inexpensive we recommend it.

## 1. Introduction

In Slovenia, the first case of LDA was diagnosed in 1969 [1]. In the last decade LDA occured most commonly in large, high-producing adult dairy cows immediately after parturition. Yearly retrospective analyses of our ambulatory records for dairy herds in region Gorenjska, Slovenia, showed significantly increased prevalence of LDA (2000 = 0.9%, 2010 = 3.7%), especially in cows after first parturition [2]. The cause of LDA in cattle is multifactorial but is related primary to feed intake before and after calving. The transition period occurring 2 weeks prepartum through 2–4 weeks postpartum is the major risk period in the etiology of LDA [3].

During 2007 to 2008 we treated 83 cows with LDA in field conditions. We used conservative treatment of LDA with rolling technique and oral electrolyte therapy. With this technique we successfully treated only 58% cows; therefore we were compelled to use another technique [4]. 

Surgical replacement is now commonly practiced, and many techniques have been devised with emphasis on avoidance of recurrence of the displacement. Because of good results as recorded in the literature and encouragement of Keith E. Sterner, the author of this method, we want to try the right paramedian abomasopexy—Grymer/Sterner model [4, 5].

## 2. Materials and Method

Since May 2009 till October 2011 109 cows from 46 farms were operated on because of LDA.

Diagnosis of the condition requires examination by inspection of the contours, palpation, ballottement, simultaneous percussion, and auscultation over both sides of the wider abdominal area. 

LDA was clinically confirmed with the following symptoms: the cow stopped eating concentrate, feed intake <50% of normal, milk yield below normal, lying down on right side, rumen rate decreased, rumen sounds present but muffed, feces scant and pasty, mild dehydration, and ping effect over left abdomen.

Percussion, using flick of the finger or a plexor, and simultaneous auscultation over an area between the upper third of the ninth and twelfth ribs of the abdominal wall commonly elicits the high-pitched tympanic sounds (pings) that are characteristic of LDA.

**Roll-and-Toggle-Pin Suture Procedure [5, 6].**

**Figure 1:**
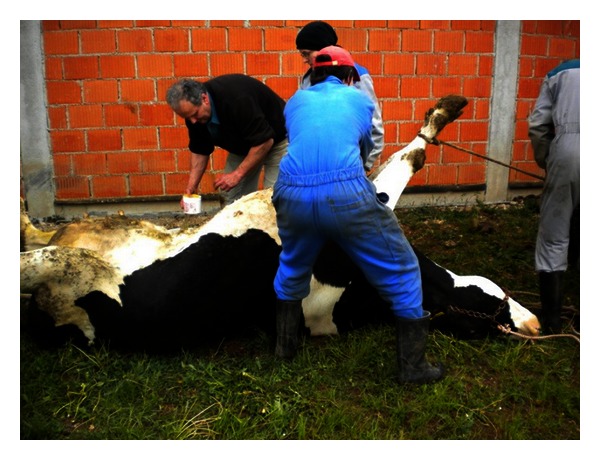
The cow is cast, restrained in right lateral recumbency, and than carefully rolled into dorsal recumbency.

**Figure 2:**
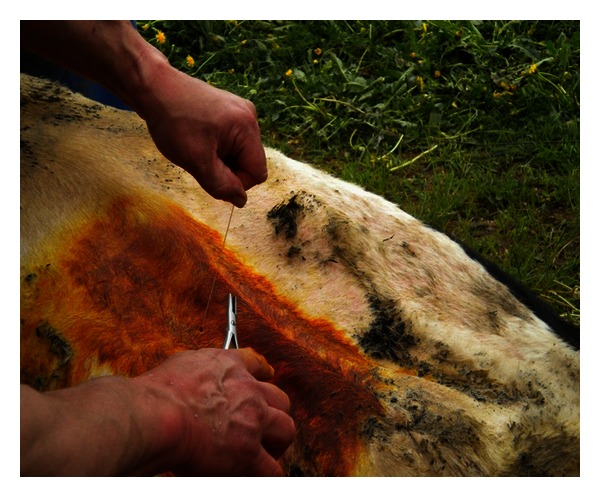
A bar suture is placed in the cannula and pushed into the abomasal lumen with the trocar. The cannula and trocar are then removed, leaving the suture in place.

The cow is cast, restrained in right lateral recumbency, and then carefully rolled into dorsal recumbency (Figure 1). Percussion and auscultation of the body wall between the umbilicus and xyphoid for detection of a “ping” reveals the location of the abomasum and the site of trocarization. In the region of the loudest “ping,” usually on the right side of the ventral midline, a trocar cannula [4] is inserted through the abdominal wall into the abomasum, avoiding the subcutaneous abdominal vein. The trocar is then removed with the cannula left in place. Perforation of the abomasum is suggested by the distinctive odor of escaping abomasal gas and confirmed by determination of a pH 2-3 in aspirated content. A bar suture is placed in the cannula and pushed into the abomasal lumen with the trocar (Figure 2). The cannula and trocar are then removed, leaving the suture in place. In a similar manner, a second suture is placed about 5 cm from the first. Prior to removal of the second cannula, abomasal gas is allowed to escape. The suture is tied loosely. The cow is rolled into left lateral recumbency and allowed to stand.

## 3. Results

Since May 2009 till October 2011 109 cows from 46 farms were operated on because of LDA. As many as 44 (40.3%) were affected with LDA after first parturition.

The analysis of successful procedure that was carried out 2 months after suture showed that 104 (95.41%) cows were cured. Only 5 (4.5%) cows died within 24 hours after surgery (2 cases of severe toxemia with hypokalemia and one case of acute abomasal hemorrhage were established).

## 4. Discussion

**Figure 3:**
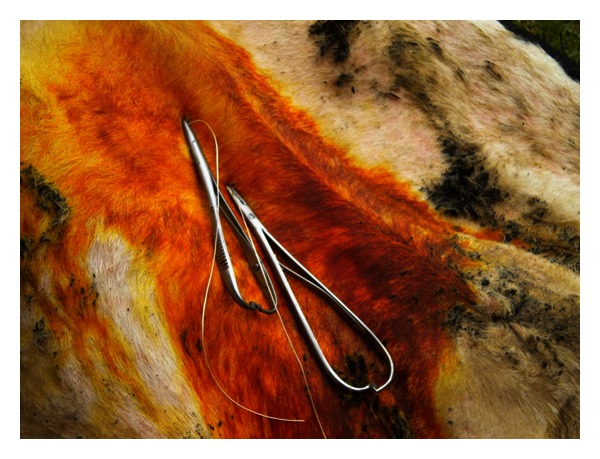
Fixation of Grymer/Sterner suture with two needle holders.

**Figure 4:**
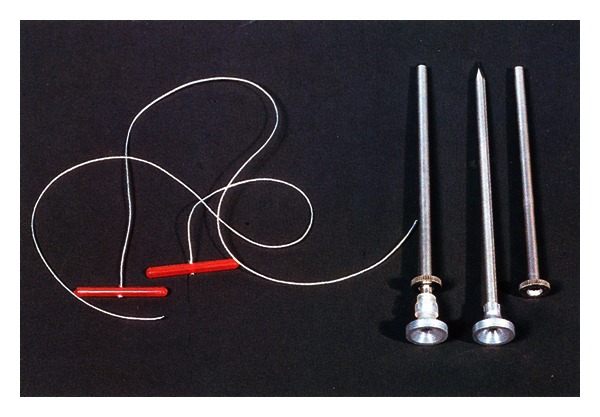
LDA Transfixation Sutures—Grymer/Sterner model.

On the basis of our observation and clinical experiences we are of the opinion that the majority of cows with LDA are lying down on the right side. Thus the pain is avoided as well as the pressure on the left side, as the distending abomasum protrudes in deep dorsal paralumbar fossa. With progressing dislocation the omasum, reticulum, and liver are also rotated to varying degrees. We also believe that the prognosis for recovering is good if the cow lies down on its left side and starts eating immediately after rolling and oral therapy. This is a good sign that abomasum is pushed into its physiological position. Our experience with LDA correction via the roll-and-toggle-pin suture according to Grymer/Sterner model showed that percutaneous abomasopexy using a bar suture is the appropriate method (Figures 3 and 4). Because roll-and-toggle-pin suture technique is rapid and inexpensive we recommend it.
